# Odyssée: protocol for a prospective cohort on psychiatric transition at age 17 in Nice, France

**DOI:** 10.1136/bmjopen-2026-120667

**Published:** 2026-08-25

**Authors:** Louise-Emilie Dumas, Simone Marchini, Eric Fontas, Julie Ribeyron, Victoria Metelkina-Fernandez, Michel Benoit, Arnaud Fernandez, Florence Askenazy

**Affiliations:** 1 Service Universitaire de Psychiatrie de l'Enfant et de l’Adolescent (SUPEA), Hôpitaux Pédiatriques de Nice CHU-LENVAL, Nice, Provence-Alpes-Côte d’Azur, France; 2 Laboratoire CoBTek, Université Côte d’Azur, Nice, Provence-Alpes-Côte d’Azur, France; 3 Child and Adolescent Psychiatry, Hopital Universitaire des Enfants Reine Fabiola, Bruxelles, Belgium; 4 Délégation à la Recherche Clinique et à l’Innovation, Centre Hospitalier Universitaire de Nice, Nice, Provence-Alpes-Côte d’Azu, France; 5 Service Universitaire de Psychiatrie, Centre Hospitalier Universitaire de Nice, Nice, Provence-Alpes-Côte d’Azur, France

**Keywords:** Child & adolescent psychiatry, PSYCHIATRY, PUBLIC HEALTH

## Abstract

**Introduction:**

The transition from child and adolescent mental health services (CAMHS) to adult mental health services (AMHS) represents a critical period of vulnerability, often marked by care discontinuities and adverse clinical outcomes. International studies have highlighted the risks associated with this transition, but local epidemiological data remain scarce in France. The Odyssée study aims to provide local data to inform the development of structured transition pathways.

**Methods and analysis:**

This prospective monocentric cohort study will be conducted at Lenval University Children’s Hospital in Nice, the only paediatric hospital providing dedicated child psychiatry emergency services in the Alpes-Maritimes department. Recruitment started in April 2026 and is ongoing at the time of manuscript submission. Study completion is anticipated in September 2028. The primary objective is to determine the annual prevalence of 17-year-olds living in Nice who attend child and adolescent psychiatry services using administrative data combining census counts from the French National Institute of Statistics and Economy Studies and hospital discharge records (*Programme de médicalisation des systèmes d’information* (PMSI)).

In parallel, a prospective cohort of 100 adolescents aged 17 years will be followed for 12 months to address secondary objectives, including clinical profiles, care trajectories, transition readiness, clinical outcomes and determinants of care continuity. Assessments will be conducted at baseline, 6 months and 12 months using validated instruments (Mini International Neuropsychiatric Interview—simplified (MINI-S), WHO Quality of Life-brief (WHOQOL-BREF), Health of the Nation Outcome Scales for Children and Adolescents (HoNOSCA), Transition Readiness and Appropriateness Measure (TRAM), Transition Related Outcome Measure (TROM)). Descriptive and multivariable analyses will be performed to identify factors associated with continuity of care. This study is expected to provide one of the first city-level estimates of psychiatric transition needs in France and contribute to the international literature on continuity of care, youth mental health trajectories and transitional service organisation.

**Ethics and dissemination:**

The study has been approved by the Comité de Protection des Personnes Sud-Ouest et Outre-Mer IV (approval number 2025-A01581-48). Written informed consent will be obtained from participants and one parent or legal guardian. Findings will be disseminated through peer-reviewed publications, scientific conferences and reports to regional health authorities.

**Trial registration number:**

NCT07068945.

**STRENGTHS AND LIMITATIONS OF THIS STUDY**

- Dual-source design combining administrative data (French National Institute of Statistics and Economy Studies/*Programme de médicalisation des systèmes d’information* (PMSI)) with prospective longitudinal follow-up.
- Standardised validated instruments (Mini International Neuropsychiatric Interview—simplified (MINI-S), WHO Quality of Life—brief (WHOQOL-BREF), Health of the Nation Outcome Scales for Children and Adolescents (HoNOSCA), Transition Readiness and Appropriateness Measure (TRAM) and Transition Related Outcome Measure (TROM)) are administered repeatedly over 12 months.
- Consecutive recruitment includes adolescents receiving outpatient, inpatient and emergency psychiatric care.
- The monocentric cohort design and sample size (n=100) may limit external generalisability.
- Attrition over 12 months may introduce bias if loss to follow-up is associated with clinical severity or care disengagement.

## Introduction

 The period between adolescence and adulthood is marked not only by physiological change but also by psychological vulnerability.^1^ Psychiatric disorders emerge before the age of 15 in 50% of cases and before the age of 25 in 75% of cases.^2 3^ In 2024, the WHO reported that 14% of individuals aged 10–19 years’ experience a mental health disorder, and suicide is the third leading cause of death among those aged 15–29 years.^4^ The transition from adolescence to adulthood is therefore a critical period both for the detection of emerging psychiatric disorders with high risk for chronicity and for ensuring continuity of care between child and adolescent mental health services (CAMHS) and adult mental health services (AMHS).^5^ This represents both an individual-level challenge, in terms of prognosis and quality of life, and a major public health issue at the societal level.^6 7^

Despite this, the organisation of care transfer from CAMHS to AMHS remains problematic. Approximately 60% of patients experience a disruption in care between child and adult psychiatry services,^8^ with an average treatment gap of about 3 months.^9^ Studies also show that 50% of patients followed in child psychiatry discontinue care altogether,^10 11^ and such discontinuity increases the risk of hospitalisation in adulthood by 20–30%.^12^ Moreover, the assessment and management of emerging psychiatric disorders in this age group, which are often transdiagnostic and non-specific, highlight the need for targeted training of healthcare professionals and adapted services.^13 14^ Barriers to access and the specific needs of this vulnerable population remain major obstacles to implementing a clear and coordinated care pathway for patients and their families.^15–18^

Transitional psychiatry refers to the practices, structures and supports designed to ensure continuity of psychiatric care between CAMHS and AMHS during the passage to adulthood.^6^ Several studies have characterised this population and highlighted the high frequency of care disruptions, the importance of early identification of young people at risk of disengagement and the need for structured protocols and dedicated teams to improve continuity and adherence.^19–23^ Importantly, discontinuity of care during this transition period has also been associated with increased risk of adverse outcomes, including hospitalisation, relapse and premature mortality, particularly in young people with severe mental disorders.^10^ Most of these studies have relied on multicentre methodologies^20 24^ or focused on specific dedicated units,^25^ but there is little evidence from local or city-level research.

Beyond organisational challenges, the transition from CAMHS to AMHS also reflects a broader developmental and epidemiological issue.^5 6^ Administrative age boundaries do not necessarily correspond to the trajectories of emerging psychiatric disorders, which often evolve across adolescence and early adulthood.^3 15^ Young people approaching the transition boundary represent a heterogeneous population, characterised by varying clinical presentations, psychosocial vulnerabilities and patterns of service use.^13 15 18^ In this context, local epidemiological studies conducted in real-world settings may contribute to the international literature by improving understanding of the needs and care trajectories of young people requiring ongoing psychiatric support during the transition to adulthood.^22 23^

Our study, Odyssée, is a local research project supported by the Regional Health Agency Provence-Alpes-Côte d’Azur. This prospective longitudinal cohort study adopts a city-level approach in Nice, linking child psychiatry administrative data with individual clinical follow-up to assess transition needs and inform specialised care pathways. The study will evaluate the transition from CAMHS to AMHS among 17-year-old adolescents living in Nice.

## Objectives

The primary objective of this study is to determine the annual prevalence of 17-year-old patients living in the city of Nice who consult specialised CAMHS child and adolescent psychiatry services, relative to the total population of 17-year-olds residing in Nice.

The secondary objectives are to:

1. Estimate the annual prevalence of 17-year-olds living in Nice who consult specialised CAMHS (Nice University Children’s Hospital(HPU Lenval)), relative to the population of minors (<18 years) in psychiatric clinical care.
2. Describe the clinical and sociodemographic profiles of a sample of 17-year-old patients living in Nice and consulting specialised CAMHS (HPU Lenval).
3. Characterise care trajectories over a 12-month follow-up period, according to the evolution of clinical management.
4. Assess the clinical course of patients during follow-up.
5. Evaluate the level of readiness for transition to adult psychiatry.
6. Identify factors associated with continuity of psychiatric care at 12 months.

Taken together, these objectives aim to generate actionable local evidence to tailor transition planning and service organisation to the specific needs of young people in Nice. Findings will inform locally adapted tools and dedicated pathways to improve continuity of care across the CAMHS–AMHS transition.

## Methods and analysis

### Study design

The study includes two components:

- An epidemiological analysis estimating the annual prevalence of 17-year-olds living in Nice who attend specialised CAMHS, using French National Institute of Statistics and Economy Studies (INSEE) census counts (population denominator) and *Programme de médicalisation des systèmes d’information* (PMSI) hospital discharge data from Lenval University Children’s Hospital (clinical numerator).
- A prospective monocentric cohort of 100 adolescents aged 17 years receiving psychiatric care at Lenval University Children’s Hospital, followed for 12 months with assessments at baseline, 6 months and 12 months.

The overall study duration is 36 months (18-month recruitment, 12-month follow-up and 6-month analysis). The overall study duration is approximately 30 months. Recruitment started on 7 April 2026 and is ongoing at the time of manuscript submission. Participant inclusion is planned over an 18-month period. Primary completion and overall study completion are both anticipated in September 2028, in accordance with the ClinicalTrials.gov, registration: NCT07068945.

### Eligibility criteria

Eligibility criteria are defined as follows:

#### Inclusion criteria

Participants must meet all of the following criteria:

- Age ≥17 and <18 years at inclusion.
- Receiving care (outpatient, inpatient or emergency) within the Child and Adolescent Psychiatry Department at Lenval University Children’s Hospital.
- Presence of a psychiatric disorder according to Diagnostic and Statistical Manual of Mental Disorders, Fifth Edition (DSM-5) criteria, irrespective of diagnostic category.
- Considered to be in a potential transition situation to adult mental health services within the following 12 months.
- Living in the municipality of Nice (postal codes 06000, 06100, 06200, 06300).
- Able to provide written informed consent, together with at least one parent or legal guardian.

#### Exclusion criteria

Participants meeting any of the following criteria will be excluded:

- Insufficient command of French preventing comprehension of study information or completion of questionnaires.
- Cognitive or sensory impairments (eg, moderate-to-severe intellectual disability, major visual impairment, severe reading difficulties) preventing valid completion of assessments.
- Legal incapacity (eg, guardianship) preventing independent consent.
- Acute or unstable clinical condition requiring urgent or intensive care incompatible with research participation (eg, severe psychiatric decompensation, immediate suicidal crisis).
- Refusal to participate by the adolescent or the parent(s)/legal representative(s).
- Any situation judged by the investigator as incompatible with 1 year follow-up (eg, imminent transfer to another region).

Withdrawal criteria

- Withdrawal of consent by the adolescent or the legal representative(s).

### Recruitment and informed consent

Eligible adolescents and their parents or legal guardians will receive written and oral information about the study from a member of the research team who is not directly involved in the patient’s clinical care. Potential participants are identified consecutively by clinicians within the Child and Adolescent Psychiatry Department during routine outpatient consultations, inpatient admissions, day hospital care or emergency psychiatric assessments. Screening logs are maintained to document the number of eligible patients approached, included or refusing participation during the recruitment period. To ensure voluntary participation, a minimum 72-hour reflection period will be respected between information and consent. At inclusion (age 17), written informed consent will be obtained from at least one parent or legal guardian, together with the adolescent’s written assent. In accordance with French regulations governing minimal-risk interventional research involving minors, parental consent remains mandatory until participants reach the age of 18 years. When participants turn 18 during follow-up, renewed written consent will be obtained to continue participation. Participation or refusal will have no impact on clinical care, and withdrawal is possible at any time without consequences.

### Study procedures

The study comprises two complementary components:

1. Prevalence study (administrative data).

Annual prevalence will be calculated for 17-year-olds living in the municipality of Nice who receive care in child and adolescent psychiatry services at the University Children’s Hospital, regardless of care setting, including inpatient admissions, day hospital care, scheduled outpatient consultations and emergency psychiatric assessments.

- Population denominator:

The number of 17-year-olds living in Nice will be obtained from the most recent INSEE census data available (2022). Given the very small year-to-year variation in population counts for this age group, the 2022 figure will be used as the reference denominator for estimating 2025/2026 prevalence.

- Clinical numerator:

Hospital utilisation data will be obtained from the PMSI system at Lenval University Children’s Hospital for the year 2024, which is the most recent complete dataset available at the time of analysis. These data will provide the number of 17-year-olds living in Nice who accessed child and adolescent psychiatry services, as well as the total number of minors (<18 years) receiving psychiatric care during 2024.

1. Longitudinal cohort study:

A cohort of 100 adolescents aged 17 years will be prospectively recruited and followed for 12 months at a University Children’s Hospital. Three study visits are planned: baseline (V0), 6 months (V1) and 12 months (V2). Follow-up assessments at V1 and V2 are conducted irrespective of whether participants have turned 18 during the study.

- Baseline (V0): After written informed consent is obtained from the adolescent and a parent/legal guardian, the participant will meet with a trained evaluator (principal investigator or delegated clinician) who will explain the study procedures, address any questions and introduce the assessment tools. This brief clinical interview (≈20–30 min) is not a medical examination and involves no clinical intervention; it may be conducted on site or via secure teleconsultation.
  Participants will then complete the baseline assessments, including:
  - Collection of sociodemographic and medical information.
  - Assessment of psychiatric symptoms and diagnoses (Mini International Neuropsychiatric Interview—simplified (MINI-S)).
  - Evaluation of quality of life (WHO Quality of Life—brief (WHOQOL-BREF)) and care needs/psychosocial difficulties (Health of the Nation Outcome Scales for Children and Adolescents (HoNOSCA)).
  - Assessment of readiness for transition (Transition Readiness and Appropriateness Measure (TRAM)).
- 6-month follow-up (V1): Assessments will include:
  - MINI-S.
  - WHOQOL-BREF.
  - HoNOSCA.
  - TRAM.
- 12-month follow-up (V2): Assessments will include:
  - MINI-S.
  - WHOQOL-BREF.
  - HoNOSCA.
  - Evaluation of transition outcomes using the Transition Related Outcome Measure (TROM).

**Figure 1 F1:**
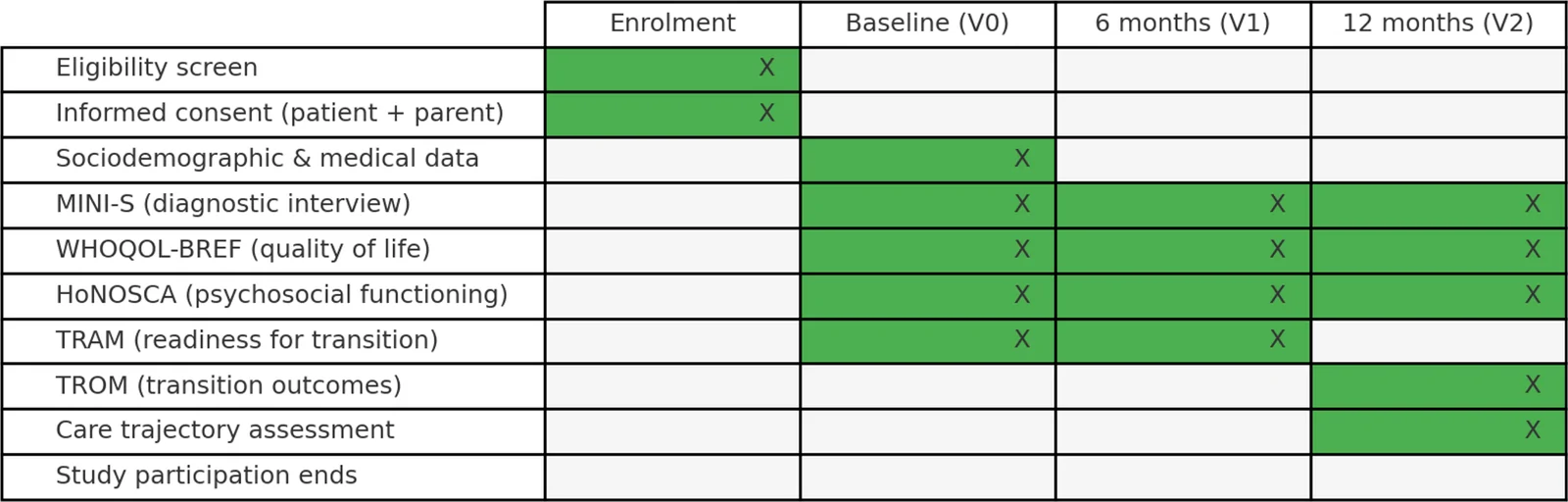
[Fig F1]SPIRIT figure—schedule of enrolment, assessments and follow-up. The table shows the schedule of study enrolment, baseline assessments and follow-up visits at 6 and 12 months. Rows indicate the study procedures and outcome measures; columns represent the study time points. An ‘X’ indicates the time point at which each procedure or assessment is conducted. HoNOSCA, Health of the Nation Outcome Scales for Children and Adolescents; MINI-S, Mini International Neuropsychiatric Interview—simplified; SPIRIT, Standard Protocol Items: Recommendations for Interventional Trials; TRAM, Transition Readiness and Appropriateness Measure; TROM, Transition Related Outcome Measure; WHOQOL-BREF, WHO Quality of Life—brief.

The schedule of enrolment, assessments and follow-up is presented in figure 1. Study participation will end after the 12-month follow-up. Patients will continue to receive routine clinical care according to current recommendations and individual clinical needs.

### Outcomes

#### Primary outcome

The primary outcome is the annual prevalence of 17-year-old patients living in the municipality of Nice who attend the child and adolescent psychiatry services at Lenval University Children’s Hospital, defined as the number of 17-year-olds consulting in 1 year divided by the total number of 17-year-olds living in the general population of Nice.

#### Secondary outcomes

1. Annual prevalence of 17-year-old patients living in the municipality of Nice who attend the child and adolescent psychiatry services (Lenval University Children’s Hospital) in 1 year, expressed as the proportion of these patients relative to the total number of minors (<18 years) consulting in the hospital during the same year (psychiatric clinical population).
2. Baseline description of patient profiles in the prospective cohort (n=100), including sociodemographic and medical characteristics.
3. Care trajectories at 12 months, categorised as:
  - Continued care in child and adolescent psychiatry.
  - Transfer to adult psychiatry.
  - Discontinuity or interruption/resumption of care during follow-up.
  - Care formally discontinued in agreement between patient and care team.
  - Lost to follow-up.
4. Clinical evolution assessed using the MINI questionnaire at baseline (M0), 6 months (M6) and 12 months (M12), focusing on status. Additional outcomes include quality of life (WHOQOL-BREF) and care needs/psychosocial difficulties (HoNOSCA) scores at the same time points.
5. Readiness and outcome of transition: measured with the TRAM at M0 and M6, and the TROM at M12.
6. Continuity of psychiatric care at 12 months, defined as ‘effective’ if the patient has a structured care plan in place or has recovered and no longer requires follow-up. This binary outcome (yes/no) will be determined by the investigator based on patient evaluation. Predictors of continuity will include prespecified baseline variables selected on clinical relevance and prior literature, including sex, living situation, psychiatric history, current diagnostic profile (MINI-S), quality of life (WHOQOL-BREF), care needs and psychosocial difficulties (HoNOSCA) and transition readiness (TRAM).

Active recontact procedures (phone/SMS/email reminders and, when relevant, parental contact) will be used to limit attrition. Participants will be considered lost to follow-up only if no contact can be re-established and no information on ongoing care can be obtained.

### Measures

#### Sociodemographic and medical data: 

Baseline information will include living situation, schooling/occupation, care pathways, psychiatric and medical history and treatment exposure. These variables are collected from clinical records and participant report to characterise the study population and explore factors associated with continuity of care during the transition period.

#### MINI-S (DSM-5; French V.1.2):

The MINI-S is a brief, structured diagnostic interview aligned with DSM-5, designed for administration by clinicians trained in DSM-5 criteria. The adult version is applicable from age 17. The instrument has established reliability and validity and has been validated in both French and English.^26 27^ In this study, the MINI-S will be administered at M0, M6 and M12 to assess current (present-state) diagnoses and symptom status. We will use diagnostic modules and summary indicators; item-level data will not be modelled in multivariable analyses

#### WHOQOL-BREF:

The WHOQOL-BREF is a validated 26-item questionnaire developed by the WHO that yields four domain scores—physical, psychological, social relationships and environment—plus two global items. Items are rated on 5-point Likert scales and transformed to 0–100 domain scores per WHO guidance.^28^ The French version has been validated and used in both general and psychiatric populations,^29^ with good psychometric properties and cross-cultural validity. The WHOQOL-BREF will be administered at M0, M6 and M12; analyses will focus on domain-level scores.

#### HoNOSCA:

HoNOSCA is a 13-item clinician-rated scale (0–4 per item; higher scores indicate greater difficulties) covering behavioural, emotional, social, somatic and functioning problems, and is commonly used to assess care needs and monitor clinical change in routine child and adolescent mental health services.^30^ A validated French self-report version (F-HoNOSCA-SR) is also available^31^ and may be used to complement clinician ratings where feasible. HoNOSCA will be administered at M0, M6 and M12; analyses will use total and selected domain subscores.

#### TRAM:

Developed within the Managing the Link and Strengthening Transition from Child to Adult Mental Healthcare in Europe (MILESTONE) programme, the TRAM is a validated multidimensional measure assessing the appropriateness and readiness for transition from child/adolescent to adult mental health services.^32 33^ It covers symptom frequency/severity, global illness impact, daily functioning impact, risk behaviours, diagnostic complexity/factors and satisfaction with care. To maximise feasibility, only the patient-reported version will be used in this study. TRAM will be administered at M0 and M6; analyses will prioritise the total readiness/appropriateness indices and a restricted set of prespecified subscales.

#### TROM:

The TROM, also validated within MILESTONE,^34^ evaluates the quality and outcomes of the transition process, including clinical status, service use/engagement and satisfaction. It will be administered at M12 (end of follow-up) to capture post-transition outcomes. Analyses will focus on the global outcome index and a limited number of predefined subscales.

Given the planned sample size (n=100), analyses will focus on composite indices and domain-level scores from each instrument, rather than item-level data. Only the most clinically and methodologically relevant variables will be retained for multivariable models to reduce the risk of over-parameterisation

### Sample size estimation

Reliable local prevalence data on 17-year-olds at the transition boundary between child and adult mental health services are currently lacking. The present study addresses this gap through an epidemiological component based on INSEE census counts and PMSI hospital discharge data.

For the prospective cohort, the target sample size (n=100) was determined a priori using a feasibility-based approach. It was informed by the annual number of 17-year-olds receiving psychiatric care at Lenval University Children’s Hospital (approximately 160–200 per year), expected consent and participation rates observed in previous local non-interventionnal studies involving human participants (Recherche Impliquant la Personne Humaine, category 3 under the French regulatory framework), and the practical feasibility of recruitment within an 18-month inclusion period. This sample size is expected to provide adequate precision for descriptive estimates of clinical and sociodemographic characteristics and to support exploratory multivariable analyses examining factors associated with continuity of care at 12 months. Given the exploratory nature of secondary analyses, effect-size assumptions for formal power calculations were not available; therefore, sample size was primarily driven by feasibility and the objectives of estimation and model exploration.

### Statistical analysis

All analyses will be conducted by a biostatistician from the Clinical Research Department of Nice University Hospital using SAS Enterprise Guide V.7.1 (SAS Institute, Cary, North Carolina, USA). A two-sided p value <0.05 will be considered statistically significant, unless otherwise specified. Assumptions underlying statistical tests will be systematically verified prior to use.

#### Descriptive analyses

 Baseline characteristics of the study population will be summarised using absolute and relative frequencies (with 95% CIs) for categorical variables, and means (±SD), medians and IQRs for quantitative variables. A flow chart will present the number of patients included and followed at each stage.

#### Primary outcome

 The annual prevalence of 17-year-old patients living in Nice who attend child and adolescent psychiatry services at Lenval University Children’s Hospital will be calculated as the ratio of the number of patients attending services to the total number of 17-year-olds in the general population of Nice, with 95% CIs.

#### Secondary outcomes

- Prevalence relative to the broader clinical population of minors (<18 years) attending Lenval psychiatry services.
- Patient profiles at baseline: descriptive statistics for sociodemographic and clinical variables.
- Care trajectories over 12 months: described using absolute and relative frequencies.
- Clinical assessments (MINI-S, WHOQOL-BREF, HoNOSCA): summarised at baseline, 6 months and 12 months; visualised using histograms and line graphs.
- Transition readiness (TRAM) and outcomes (TROM): summarised with means, SD, medians and IQRs; visualised by domains.

#### Continuity of care analyses

 Continuity of psychiatric care at 12 months (binary outcome: defined care plan vs no defined plan) will be examined using logistic regression. Univariate logistic models will first be applied; variables associated with continuity of care at p<0.20 will be considered for inclusion in a multivariable logistic regression model. Results will be reported as ORs with 95% CIs. Collinearity will be assessed prior to multivariable modelling. Given the planned sample size (n=100), multivariable models will be restricted to a limited number of prespecified variables selected on the basis of clinical relevance and previous literature, including sex, living situation, psychiatric history, baseline diagnostic profile (MINI-S), quality of life (WHOQOL-BREF), care needs and psychosocial difficulties (HoNOSCA) and transition readiness (TRAM), in order to reduce the risk of overfitting and ensure robustness of results.

#### Missing data

No imputation of missing data is planned. Loss to follow-up will be characterised by comparing baseline sociodemographic and clinical variables between participants who complete the study and those lost to follow-up. Sensitivity analyses will be conducted to assess the potential impact of missing data on the main outcomes.

### Data management

Study data will be collected using a dedicated case report form (CRF), developed in collaboration with the study sponsor’s clinical research associate and the data manager of the Clinical Research and Innovation Department (DRCI) of Nice University Hospital, under the supervision of the principal investigator. Data will be entered directly into an electronic CRF (e-CRF) created with Research Electronic Data Capture, which will be configured and maintained by the DRCI.

Investigators, supported by the study clinical research associate, will be responsible for accurate data entry and ongoing verification. Access rights to the e-CRF will be restricted according to user roles. Data quality will be ensured through programmed consistency checks and monitoring procedures, with any queries resolved in collaboration with investigators.

At the end of the study, the database will be locked (‘frozen’) once validation checks are complete and signed off by the principal investigator, the data manager, and the study statistician. The locked dataset will then be transferred for statistical analysis.

### Ethics and dissemination

The study received ethical approval from the Comité de Protection des Personnes Sud-Ouest et Outre-Mer IV (CPP, French research ethic committee), approval number 2025-A01581-48. In accordance with French regulations, the study is classified as a minimal-risk interventional research project. Written informed consent will be obtained from all participants and their legal guardians prior to inclusion. Participant data will be collected and processed in accordance with applicable confidentiality and data-protection regulations, including the General Data Protection Regulation. Data will be pseudonymised prior to analysis, stored on secure servers with restricted access and retained for the legally required duration. The trial is registered at ClinicalTrials.gov (NCT07068945). Study findings will be disseminated through peer-reviewed publications, presentations at national and international scientific conferences and reports to relevant health authorities.

## Discussion

This study has several strengths. Its prospective longitudinal design focuses on a critical developmental stage, while the use of validated assessment tools (MINI-S, WHOQOL-BREF, HoNOSCA, TRAM, TROM) ensures methodological rigour and comparability with international work. By combining administrative data (INSEE, PMSI) with individual clinical follow-up, the study will provide a comprehensive picture of transition needs across all public child psychiatry services in a large French city.

Importantly, the study will address a gap in French epidemiology by providing the first city-level estimate of the annual prevalence of 17-year-olds accessing specialised CAMHS. This is essential for quantifying service needs at the transition boundary and informing resource planning. Although based on PMSI 2024 data, it offers a robust 1-year snapshot based on routinely collected administrative records; year-to-year variability cannot be assessed within the present dataset.

Some limitations must be acknowledged. The single-centre design and modest sample size (n=100) may restrict generalisability and limit the power of multivariable analyses. Anticipated loss to follow-up at 12 months also reflects the challenges inherent in transitional psychiatry.^21^ In addition, the absence of a control group or multicentre setting constrains external comparisons.

Although young people and parents were not directly involved in the design of this protocol, their needs and concerns routinely observed in clinical practice strongly informed the choice of outcomes and follow-up procedures. Adolescents and families frequently express anxiety regarding the transition to adult services, including fears of losing established therapeutic relationships, uncertainty about the functioning of adult-oriented services and concerns about changes in parental involvement after the age of 18. Clinicians also observe that periods of educational, social or familial instability may increase the risk of disengagement from care, particularly among young people with evolving or non-stabilised psychiatric presentations. These observations are broadly consistent with the international literature^5 6 22^ and contributed to our decision to include measures of transition readiness, continuity of care, quality of life and care trajectories. Future iterations of the project will integrate formal patient and public involvement to further strengthen the relevance and acceptability of transition pathways.

Despite these limitations, the Odyssée project is expected to yield valuable insights. Clinically, it should help identify patients at risk of disengagement and inform structured interventions to support continuity of care. Organisationally, beyond providing descriptive epidemiological data, the study is intended to serve as a foundation for building a dedicated care pathway and specialised team to address the specific needs of young people during the transition to adult psychiatry. At a broader level, the findings will inform regional planning and may serve as a pilot model for replication in other regions, contributing to the international literature on transitional mental healthcare.^35 36^
