# Supplementary material for: Odyssée: protocol for a prospective cohort on psychiatric transition at age 17 in Nice, France
Source: BMJ Open. 2026 Aug 25;16(8):e120667. doi: 10.1136/bmjopen-2026-120667 (PMC13536005; doi:10.1136/bmjopen-2026-120667)
Supplement: Supplementary file 1 [file bmjopen-2026-120667.reviewer_comments.pdf]

PEER REVIEW HISTORY

BMJ Open publishes all reviews undertaken for accepted manuscripts. Reviewers are asked to complete a checklist review form and are provided with free text boxes to elaborate on their assessment. These free text comments are reproduced below.

ARTICLE DETAILS

Title (Provisional)

Odyssée: protocol for a prospective cohort on psychiatric transition at age 17 in Nice, France

Authors

Dumas, Louise-Emilie; Marchini, Simone; Fontas, Eric; Ribeyron, Julie; Metelkina-Fernandez, Victoria; Benoit, Michel; FERNANDEZ, Arnaud; Askenazy, Florence

---

VERSION 1 - REVIEW

**Reviewer**	**1**
**Name**	**Di Simplicio, Martina**
**Affiliation**	**Imperial College London**
**Date**	**18-May-2026**
**COI**	**None**

This paper presents the protocol for an interesting study aiming to identify the prevalence and characteristics of young people transitioning from children to adult mental health services in a municipality in France. Strengths are well described in terms of combining healthcare and social care / education datasets with a research cohort defined for this project. Transitions are an important area of clinical need and service development as services do not map onto the epidemiology and presentation of youth mental health retaining the traditional divide at 18-years-old of age.

My main comment is that the paper needs some broader contextualisation to add to the international literature both in the introduction and discussion. Given the scarce research in this area, other than benefitting service planning in Nice, how will this study add to our knowledge of young people's needs and epidemiology of those needing adult mental health care?

Minor comments:

- in the Objectives I would recommend changing the term "hospital-based" child and adolescent psychiatry services to "specialist", as in certain contexts hospital-based may be misinterpreted for inpatients only;

- please clarify French legislation around the age for consent taking (e.g. in the UK 16-year-olds do not need parental consent to take part in research);

- in the study procedures under "Clinical numerator" it would be interesting to know if more specific data around service type and service utilisation will be collected (e.g. what are the services / teams that most frequently refer young people to adult services among CAMHS? what type of support have young people received in CAMHS before going to adult services and how might that impact on their trajectories?);

- in the statistical analysis under "Continuity of Care analyses" please clarify what does a 'defined care plan' mean; it would also be preferable to list which prespecified variables the authors are planning to use in the multivariate models or what will guide their choice of variables;

- in the Discussion, please give some examples of the needs and concerns of young people and carers that have been observed in clinical practice and how these differ (if they do) from the literature, as well as how the authors considered them in designing the study.

VERSION 1 - AUTHOR RESPONSE

REVIEWER 1: Dr Martina Di Simplicio, Imperial College London

We sincerely thank Dr Martina Di Simplicio for her thoughtful and constructive review. We greatly appreciated her positive assessment of the study and her insightful comments, which helped us better articulate the broader scientific relevance of our work and improve the methodological clarity of the protocol.

<u>Main comment:</u> My main comment is that the paper needs some broader contextualisation to add to the international literature both in the introduction and discussion. Given the scarce research in this area, other than benefitting service planning in Nice, how will this study add to our knowledge of young people's needs and epidemiology of those needing adult mental health care?

<u>Response:</u> Thank you for this important observation. We agree that the initial version of the manuscript primarily emphasised the implications of the study for local service planning. We have therefore strengthened both the Introduction and the Discussion to better position ODYSSÉE within the international literature on youth mental health and psychiatric transition.

Specifically, we now emphasise that the transition from CAMHS to AMHS represents not only an organisational challenge but also a developmental and epidemiological issue. We highlight that administrative age boundaries do not necessarily align with the trajectories of emerging psychiatric disorders, and that young people approaching the transition boundary constitute a heterogeneous population characterised by varying clinical presentations, psychosocial vulnerabilities and patterns of service use. We further underline that local epidemiological studies conducted in real-world settings may contribute to the international literature by improving understanding of the needs

and care trajectories of young people requiring ongoing psychiatric support during the transition to adulthood.

<u>Changes in manuscript:</u>

Introduction: *"Beyond organisational challenges, the transition from CAMHS to AMHS also reflects a broader developmental and epidemiological issue (5,6). Administrative age boundaries do not necessarily correspond to the trajectories of emerging psychiatric disorders, which often evolve across adolescence and early adulthood (3,15). Young people approaching the transition boundary represent a heterogeneous population, characterised by varying clinical presentations, psychosocial vulnerabilities and patterns of service use (13,15,18). In this context, local epidemiological studies conducted in real-world settings may contribute to the international literature by improving understanding of the needs and care trajectories of young people requiring ongoing psychiatric support during the transition to adulthood (22,23)."*

Discussion: *"Although young people and parents were not directly involved in the design of this protocol, their needs and concerns routinely observed in clinical practice strongly informed the choice of outcomes and follow-up procedures. Adolescents and families frequently express anxiety regarding the transition to adult services, including fears of losing established therapeutic relationships, uncertainty about the functioning of adult-oriented services, and concerns about changes in parental involvement after the age of 18. Clinicians also observe that periods of educational, social or familial instability may increase the risk of disengagement from care, particularly among young people with evolving or non-stabilised psychiatric presentations. These observations are broadly consistent with the international literature (5,6,22) and contributed to our decision to include measures of transition readiness, continuity of care, quality of life and care trajectories. Future iterations of the project will integrate formal patient and public involvement to further strengthen the relevance and acceptability of transition pathways."*

<u>Minor comment 1:</u> In the Objectives I would recommend changing the term "hospital-based" child and adolescent psychiatry services to "specialist", as in certain contexts hospital-based may be misinterpreted for inpatients only.

<u>Response:</u> Thank you for this suggestion. We agree that the term "specialist" more accurately reflects the scope of services considered and avoids potential misunderstanding.

<u>Changes in manuscript:</u> The terminology "*hospital-based child and adolescent psychiatry services"* was replaced by *"specialist child and adolescent mental health services"* throughout the manuscript, including in the Objectives section.

<u>Minor comment 2:</u> Please clarify French legislation around the age for consent taking (e.g. in the UK 16-year-olds do not need parental consent to take part in research).

Response: Thank you for highlighting this important contextual difference. We have clarified the French legal framework governing research involving minors. In France, for minimal-risk interventional research involving minors, written consent from at least one parent or legal guardian remains mandatory until the participant reaches 18 years of age. Participants who turn 18 during follow-up are subsequently re-consented as adults.

Changes in manuscript: Recruitment and informed consent section:

"In accordance with French regulations governing minimal-risk interventional research involving minors, parental consent remains mandatory until participants reach the age of 18 years."

Minor comment 3: In the study procedures under "Clinical numerator" it would be interesting to know if more specific data around service type and service utilisation will be collected (e.g. what are the services / teams that most frequently refer young people to adult services among CAMHS? what type of support have young people received in CAMHS before going to adult services and how might that impact on their trajectories?).

Response: Thank you for this valuable suggestion. We agree that a more detailed characterisation of service utilisation and referral pathways would provide important insights into transition processes. Within the scope of the present study, service utilisation data include the main care settings accessed within CAMHS (outpatient consultations, inpatient admissions, day hospital care and emergency psychiatric assessments). However, detailed analyses of referring teams and specific interventions preceding transition were not included in the original protocol and fall beyond the objectives of this study. We have acknowledged the relevance of these aspects as important avenues for future research.

Minor comment 4: In the statistical analysis under "Continuity of Care analyses" please clarify what does a 'defined care plan' mean; it would also be preferable to list which prespecified variables the authors are planning to use in the multivariate models or what will guide their choice of variables.

Response: Thank you for this helpful suggestion. We clarified the operational definition of continuity of care and further specified the variables considered in the multivariable analyses.

Changes in manuscript:

Continuity of care outcome: *"Continuity of psychiatric care at 12 months, defined as 'effective' if the patient has a structured care plan in place or has recovered and no longer requires follow-up."*

Statistical analyses: *"Given the planned sample size (n=100), multivariable models will be restricted to a limited number of prespecified variables selected on the basis of clinical relevance and previous literature, including sex, living situation, psychiatric history, baseline diagnostic profile (MINI-S), quality of life (WHOQOL-BREF), care needs*

and psychosocial difficulties (HoNOSCA), and transition readiness (TRAM), in order to reduce the risk of overfitting and ensure robustness of results."

Minor comment 5: In the Discussion, please give some examples of the needs and concerns of young people and carers that have been observed in clinical practice and how these differ (if they do) from the literature, as well as how the authors considered them in designing the study.

Response: Thank you for this valuable comment. We expanded the Discussion to provide concrete examples of concerns frequently expressed by young people and their families in clinical practice and to explain how these observations informed the design of the study. We also clarified that these observations are broadly consistent with the international literature.

Changes in manuscript: The Discussion section was revised as detailed above to include examples of concerns regarding loss of therapeutic relationships, uncertainty about adult services, changing parental involvement after age 18, and the risk of disengagement from care during periods of psychosocial instability.

We would once again like to thank the Editors and Reviewer for their careful evaluation of our manuscript and for their constructive suggestions. We believe that these revisions have substantially strengthened the clarity, methodological transparency, and scientific positioning of our protocol.

We hope that the revised version satisfactorily addresses all comments and concerns, and we remain at your disposal should any further clarification be required.

VERSION 2 - REVIEW

**Reviewer**	**1**
**Name**	**Di Simplicio, Martina**
**Affiliation**	**Imperial College London**
**Date**	**15-Jul-2026**
**COI**	

Thank you for considering my suggestions and comments and revising the manuscript accordingly. I don't have any further comments.
